# Morphometric criteria to differentiate *Drosophila suzukii* (Diptera: Drosophilidae) seasonal morphs

**DOI:** 10.1371/journal.pone.0228780

**Published:** 2020-02-06

**Authors:** Anh K. Tran, W. D. Hutchison, Mark K. Asplen

**Affiliations:** 1 Department of Entomology, University of Minnesota, Saint Paul, Minnesota, United States of America; 2 Natural Sciences Department, Metropolitan State University, Saint Paul, Minnesota, United States of America; Universite du Quebec a Chicoutimi, CANADA

## Abstract

Temperate insect species often enter diapause in preparation for overwintering. One such species is the invasive vinegar fly, *Drosophila suzukii* (Matsumura), which has seasonal polymorphisms, considered winter and summer morphs. To date, the morphs have been differentiated by color and size with winter morphs typically being darker and larger compared to summer morphs. ‘Dark’ and ‘large’ are subjective, however, and standardizing an identification process can ensure that the morph of interest is being accurately characterized. The goal of our research was to investigate a quantitative method to distinguish between *D*. *suzukii* morphs based on body and wing size. We reared winter and summer morph *D*. *suzukii* in the laboratory using standard procedures, and measured wing length, wing width, and hind tibia length. Additionally, we collected field *D*. *suzukii* to document the seasonal phenology of the morphs in Minnesota based on our model’s cutoff criteria. A classification and regression tree analysis were used to determine which metrics would be best for predicting field-caught *D*. *suzukii* morphs. Using laboratory-reared flies as our known morphs for the training data in the classification model we developed classification trees based on wing length and the ratio of wing length to hind tibia length. The frequency of winter and summer morphs present in the field varied based on which classification tree was used. Nevertheless, we suggest ratio of wing length to hind tibia length as the most robust criteria for differentiating *D*. *suzukii* morphs because the ratio accounts for the size variability between laboratory-reared and field-caught flies and the error rate of misclassification is reduced to 0.01 for males. The results from this work can aid in future *D*. *suzukii* research by allowing scientists to objectively differentiate the morphs, and thereby improve our understanding of the biology and phenology of seasonal morph dynamics.

## Introduction

Ectotherms face survival challenges when inhabiting regions that experience seasonal weather changes. In preparation for cold stress, many insect species are adapted to maintain homeostasis in cooler temperatures by entering a diapause state [1]. Within the diapause state, reduced metabolism and biochemical alterations affect the insect’s biology, behavior, and morphogenesis [1].

One such diapausing species that exhibits this phenomenon is the vinegar fly, *Drosophila suzukii* (Matsumura), commonly known as spotted-wing drosophila. Native to east and southeast Asia, *D*. *suzukii* is an important economic pest of soft-skinned fruits (e.g., strawberries, raspberries, blueberries) and stone fruits (e.g. cherries, peach) [2–6]. *Drosophila suzukii* invaded the continental United States in 2008 and rapidly spread throughout the country [7], and has quickly become a global pest [2,8]. Given its recent global spread, a remarkable amount of research has been published to improve understanding the biology of *D*. *suzukii*, such as host range and preferences [3,9,10], and management strategies including chemical control [11–14], biological control [15–18], physical exclusion through netting [19–21], and enhanced monitoring techniques [12,22,23]. However, to develop improved management strategies, a broader understanding of *D*. *suzukii* biology, phenology and overwintering strategies is needed [2,24].

Recent research has shown that *D*. *suzukii* can tolerate temperate regions by producing seasonal morphs via polyphenism [25,26]. For *D*. *suzukii*, a decrease in photoperiod and temperature will produce ‘winter’ morphs, which have been described as larger in body size, wing size, and darker in pigmentation in comparison to summer morphs [25,27]. In laboratory settings, these environmental cues can be simulated to produce winter morphs for experimental purposes; for example, winter morphs can be generated by placing eggs and larvae produced by summer morphs at a constant temperature between 10–15°C and a photoperiod between 10:14 (L:D) hours and 12:12 (L:D) hours [26–29].

Winter morph *D*. *suzukii* are more cold-tolerant than summer morphs, which would allow flies to persist in temperate regions [26,29,30]. Researchers have documented *D*. *suzukii* seasonal population dynamics across different regions [12,31–34] and in several crops over time [35–37]. In addition, research describes the reproductive status and ovary maturity levels of *D*. *suzukii* in temperate regions shows that reproduction ceases, or is greatly reduced in cooler months [33,38–40]. Given these results, and in most temperate regions of the U.S., it is generally assumed that *D*. *suzukii* overwinters as an adult winter morph [41–43]. There is a paucity of research investigating the phenology or abundance of winter morphs in the field, particularly in the extreme northern range of the distribution, perhaps due to the difficulty of differentiating winter and summer morphs.

Current methods for differentiating winter and summer morphs have focused primarily on melanization changes that occur during autumn at northern latitudes or temperate regions [41,44]. When rearing different morphs in the lab, Shearer et al. [25] noted that winter morph males and females had a continuous dark pigmentation on the third and fourth abdominal segment, respectively, that was completely filled. This method has been utilized to determine the seasonal phenology of winter morphs caught in the field [27,33,44]. However, there were some challenges to using color as this was the only metric for identifying winter and summer morphs. Guédot et al. [44] mentioned that morph determination for some field-collected *D*. *suzukii* was inconclusive because selected specimens were bloated, bleached or unclear. Panel et al. [33] also had difficulty assigning individuals as winter or summer, and created an intermediate category.

A quantitative method to differentiate winter and summer morphs is needed. Relying solely on color is difficult because *D*. *suzukii* is usually collected using trapping systems where specimens drown in a liquid solution and may remain in the trap under a variety of environmental conditions prior to collection and transport to the lab [12,21,44]. Thus, the trapping system and delays in collection and processing can damage the adults making color identification difficult. The goal of this study was to develop a quantitative method for identifying *D*. *suzukii* winter and summer morphs based on well-known morphological metrics.

The morphometrics we chose were wing length, wing width, and hind tibia length because these are sclerotized body parts. Additionally, a ratio of wing length to hind tibia length was used because *D*. *suzukii* size can vary based on diet [27,33], and because temperature-induced phenotypic plasticity in wing size and shape is well known in *Drosophila* broadly, and *D*. *suzukii* specifically [45–47]. By using a ratio of wing length to hind length, we correct for potential wing size variation of field-caught versus laboratory-reared *D*. *suzukii* that may be associated with body size. We used hind tibia length because this is a standard measurement of body size outside of the flight motor apparatus [48–51].

Here we examine and report specific morphometrics that would be the best indicators of winter and summer morphs. Additionally, we apply the morphometric approach to distinguish morphs and document the seasonal phenology of field-collected *D*. *suzukii* by morph in Minnesota, near the northern limits to its range.

## Materials and methods

### Insects

Studies were conducted using laboratory-reared and field-collected *D*. *suzukii* adults. A laboratory *D*. *suzukii* colony was established in the laboratory by collecting infested raspberry fruit in 2016, produced at the University of Minnesota Outreach, Research and Education (UMORE) Park (44.7279°N, 93.0968°W) in Rosemount, MN and maintained in the Department of Entomology, University of Minnesota, St. Paul, MN. Protocols and methods for rearing *D*. *suzukii* summer and winter morphs were performed as described by Stephens et al. [26]. Summer morphs were reared in narrow polystyrene vials with foam plugs (Genesee Scientific, San Diego, CA). Each vial contained approximately 5ml of an agar-yeast-cornmeal diet and a strip of filter paper to reduce mortality from condensation. Vials were stored at 22 ± 1°C, a photoperiod of 16:8 (L:D) hours and relative humidity of 60 ± 10%. Adult winter morph *D*. *suzukii* were produced by placing vials with 1 to 3-day-old eggs from summer morphs and into a growth chamber (Percival Scientific Inc., Perry, IA) set at 10 ± 1°C, a photoperiod of 12:12 (L:D) hours and relative humidity of 70 ± 10%. At 10°C, winter morphs typically eclose within approximately 56 days. Adult summer and winter morph flies that were approximately 2-days-old (from eclosion) were used for the study.

Field populations of adult *D*. *suzukii* were collected throughout the 2017 and 2018 growing seasons, in the metropolitan area of Minneapolis-St. Paul, MN. Trap sites included the UMORE Park, a vineyard near Hastings (44.6855°N, 92.8717°W), a fresh market berry farm near Forest Lake (45.2304°N, 92.8932°W), and two mixed-berry and vegetable farms located near Andover (45.0833°N, 93.8787°W) and Waverly (45.2688°N, 93.3545°W). Permission was granted from each landowner to conduct experiments on their property. In 2018, traps were not maintained at the Andover site. In both years at each site, two commercial trapping systems were used: Pherocon^®^ SWD (Trécé Inc., Adair, OK) and Scentry (Scentry Biologicals, Inc., Billings, MT). Pherocon^®^ SWD traps consisted of a Pherocon ^®^ SWD lure and apple cider vinegar with a drop of liquid soap to lower surface tension. The Scentry trap consisted of a Scentry SWD lure and water with a drop of liquid soap, to lower surface tension. At each participating location, traps were monitored weekly to remove the contents, and transfer samples to the laboratory for processing, identification, and measurement. In 2017, traps were deployed from 15 May to 31 August except at UMORE Park where traps were taken down on 5 December. In 2018, traps were deployed from 14 May to 25 September except at UMORE Park where traps were taken down on 11 November. Lures were replaced approximately once per month and liquids (apple cider vinegar or water) were replaced weekly. As temperatures approached 0°C, the apple cider vinegar and water were replaced with propylene glycol.

### Morphometric measurements

**Fig 1:**
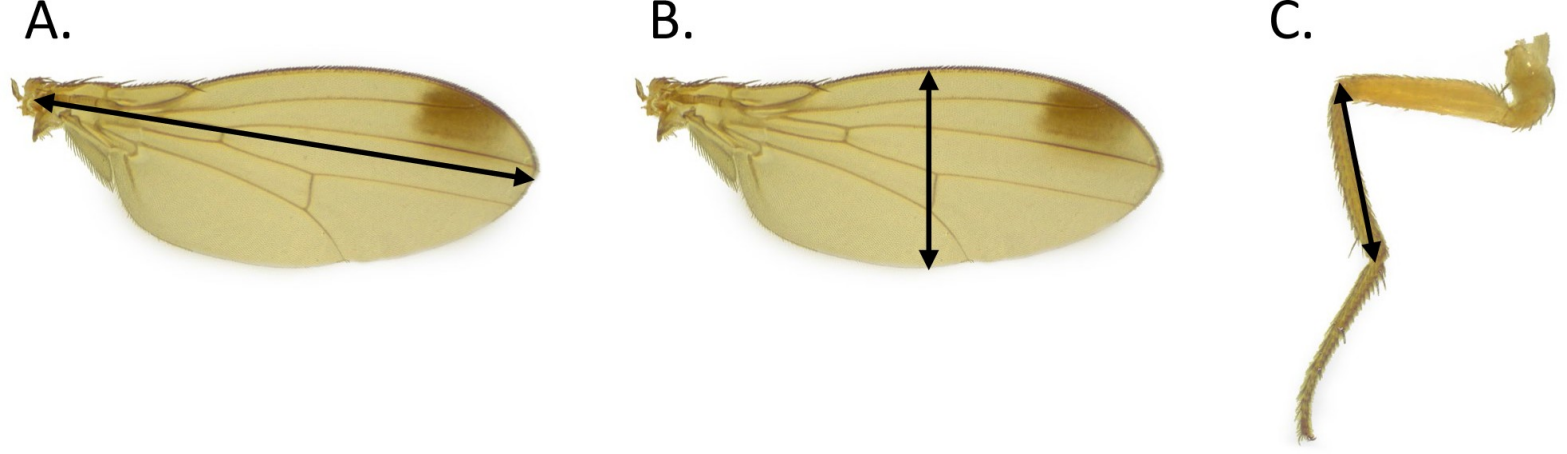
**Location of measurements for *D*. *suzukii*:** wing length (A), wing width (B), and hind tibia length (C).

Individual flies from laboratory colonies or field sites were initially placed under a stereo microscope (Leica EZ4 W, Leica Microsystems, Wetzlar, Germany) with 8x – 35x magnification. The wings and hind legs were removed from the thorax by dissecting individuals on a microscope slide with Corning^®^ Dulbecco’s phosphate-buffered saline (DPBS), 1X with calcium and magnesium solution (Mediatech Inc., Manassas, VA). Dissected contents were left on the microscope slide. Wing length, wing width and hind tibia length were measured using a compound light microscope (Leica DM500 and Leica ICC50 W, Leica Microscosystems, Wetzlar, Germany) with 4x – 100x magnification and a calibrated reticle. Measurements were only recorded from one wing and leg per fly. Wing length was measured from the base of the wing to the apex of the R4 + 5 vein (Fig 1A). Wing width was measured from the costal margin to the posterior edge following the medial cubital cross vein (Fig 1B) and the longest dimension of the hind tibia was measured (Fig 1C). In addition, the sex of each individual was recorded.

### Data analysis

A classification and regression tree analysis were used to determine the best discriminatory criteria to classify *D*. *suzukii* morphs. A classification and regression tree analysis is a non-parametric modeling approach that creates tree models by continuously splitting the data into homogenous groups based on categorical or continuous predictor variables [52,53].

In addition to descriptive statistics, data were analyzed using the package ‘rpart’ [54] via the ‘gini index’ for splitting and ‘class’ as the method in R version 3.5.2 [55] and RStudio Desktop version 1.1.463 [56]. Adult *D*. *suzukii* females are, on average, larger than males; therefore, a classification tree was constructed for each sex [57]. For the construction of the classification trees, *D*. *suzukii* morph (winter/summer) was the response variable and the predictor variables included wing length, wing width, and hind tibia length. A transformed ratio of wing length to hind tibia length was used in the classification tree. The transformation was calculated by taking the linear regression of female and male laboratory-reared known summer morphs and using the following equation adapted from Albrecht et al. [58]:

$$Y_{\mathrm{adj}}=\left(Y-\alpha \right)/X$$ Eq 1

where the expected value of Y_(adj)_ is the transformed ratio, Y is wing length, α is the intercept of the regression line and X is hind tibia length. Laboratory-reared *D*. *suzukii* were used for construction of the trees because morphs were known with certainty. To construct a classification tree, the data are split into training and validation sets. The training data set is used to generate the model, and the validation data set is used to evaluate the model’s performance. For our classification tree, 70% of the data were used for training and 30% of the data were used for validation. This method was then bootstrapped 500 times. The results from the simulation were averaged to obtain the final classification tree criteria and then applied to field-collected *D*. *suzukii* to determine the phenology of each morph.

## Results

### Descriptive statistics

Winter morph *D*. *suzukii* males, obtained from the laboratory colony, were on average larger than summer morphs males for all recorded metrics (wing length: *t* = 22.98; wing width: *t* = 8.96; hind tibia: *t* = 11.37; df = 90, *P* < 0.001, Table 1). Likewise, winter morph females were larger than the corresponding summer morph females (wing length: *t* = 23.36; wing width: *t* = 18.92; hind tibia: *t* = 9.52; df = 95, *P* < 0.001, Table 1). *Drosophila suzukii* females, for both morphs combined, were on average larger than males; this was evident for both laboratory (wing length: *t =* 5.39; wing width: *t* = 5.99; hind tibia: *t* = 8.00; df = 187, *P* <0.001) and field populations (wing length: *t* = 8.90; wing width: *t* = 8.46; hind tibia: *t =* 5.22; df = 238, *P* < 0.001 Table 1). Field-caught *D*. *suzukii* were on average larger than the laboratory-reared flies, but the mean wing length, wing width and hind tibia length were within range of the mean winter and summer morphs size range (Table 1). Additionally, the morpho-metrics for a majority of field-caught individuals were representative of the laboratory-reared *D*. *suzukii* (S1 Fig).

**Table 1:** Measurements of *D*. *suzukii* body and wing size. Mean (+/- SEM) of wing length, wing width and hind tibia length (mm) for laboratory-reared known morphs (winter and summer) of *Drosophila suzukii*, and field caught flies across the season.

	Female				Male			
Type	N	Wing Length	Wing Width	Hind Tibia Length	N	Wing Length	Wing Width	Hind Tibia Length
Winter	41	2.97 ± 0.020^a^*	1.23 ± 0.011^a^	0.80 ± 0.005^a^	42	2.69 ± 0.018^a^	1.08 ± 0.025^a^	0.75 ± 0.003^a^
Summer	56	2.39 ± 0.015^b b^	0.99 ± 0.007^b^	0.73 ± 0.004^b^	50	2.15 ± 0.015^b^	0.87 ± 0.006^b^	0.68 ± 0.004^b^
Laboratory	97	2.63 ± 0.032^A^**	1.09 ± 0.013^A^	0.76 ± 0.005^A^	92	2.40 ± 0.030^B^	0.96 ± 0.017^B^	0.71 ± 0.005^B^
Field	116	2.80 ± 0.022^A^ *	1.13 ± 0.009^A^	0.78 ± 0.005^A^	123	2.53 ± 0.018^B^	1.01 ± 0.008^B^	0.74 ± 0.005^B^

* Lowercase: Comparison of laboratory-reared winter and summer morphs within sex.

** Uppercase: Comparison of female and male laboratory-reared and field-collected *D*. *suzukii*.

Means followed by different letters represent significant differences, based on Student’s t-test (*P* <0.001).

### Classification trees

**Fig 2:**
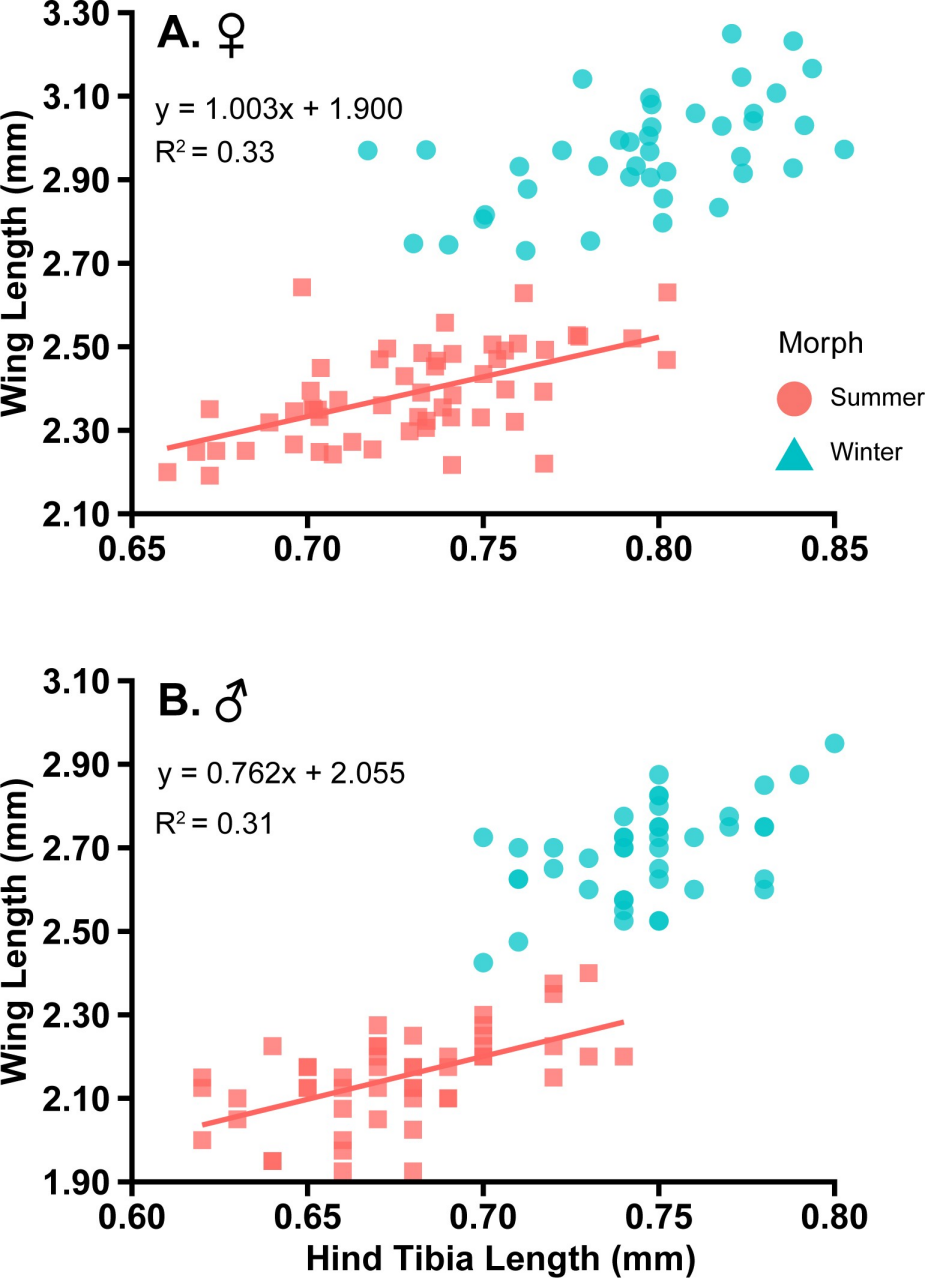
**Laboratory-reared mean wing and hind tibia lengths (mm) for known female (A) and male (B) winter and summer morphs of *D*. *suzukii*.** The linear regression equation is based on summer morphs, which was used for the ratio transformation.

Data from 97 and 92 females and males, respectively, were used to build the classification trees (Table 2). Of the three body measurements (i.e., wing length, wing width and hind tibia length), wing length was the best predictor variable for classifying winter and summer morphs from laboratory colonies. However, using the transformed ratio of wing length to hind tibia length had at a higher level of accuracy for predicting winter and summer morphs in comparison to only using wing length (Table 2). The transformed ratio utilizes the intercept of summer morphs. The intercept for laboratory-reared summer morphs was 1.003 and 0.762 for females and males, respectively (Fig 2 and S2 Fig).

**Table 2:** Summary results using wing length or the transformed ratio of wing length to hind tibia to differentiate winter and summer morphs for females and males.

	Average statistics for 500 classification simulations														
	Wing length								Ratio						
	Cutoff value (mm)				Error (%)				Cutoff value				Error%		
Sex	Mean	Min	Max		Mean	Min	Max		Mean	Min	Max		Mean	Min	Max
Female	2.69	2.63	2.73		0.10	0.00	10.35		2.17	2.12	2.22		0.10	0.00	6.90
Male	2.42	2.35	2.46		1.82	0.00	10.71		2.31	2.28	2.34		0.01	0.00	3.57

The classification model was built using measurements from laboratory-reared known morphs of *D*. *suzukii*, where 70% of the data were used for training and 30% of the data were used for validation. This process was bootstrapped 500 times and the results were averaged. For the ratio cutoff value, the data were transformed using Eq 1.

**Fig 3:**
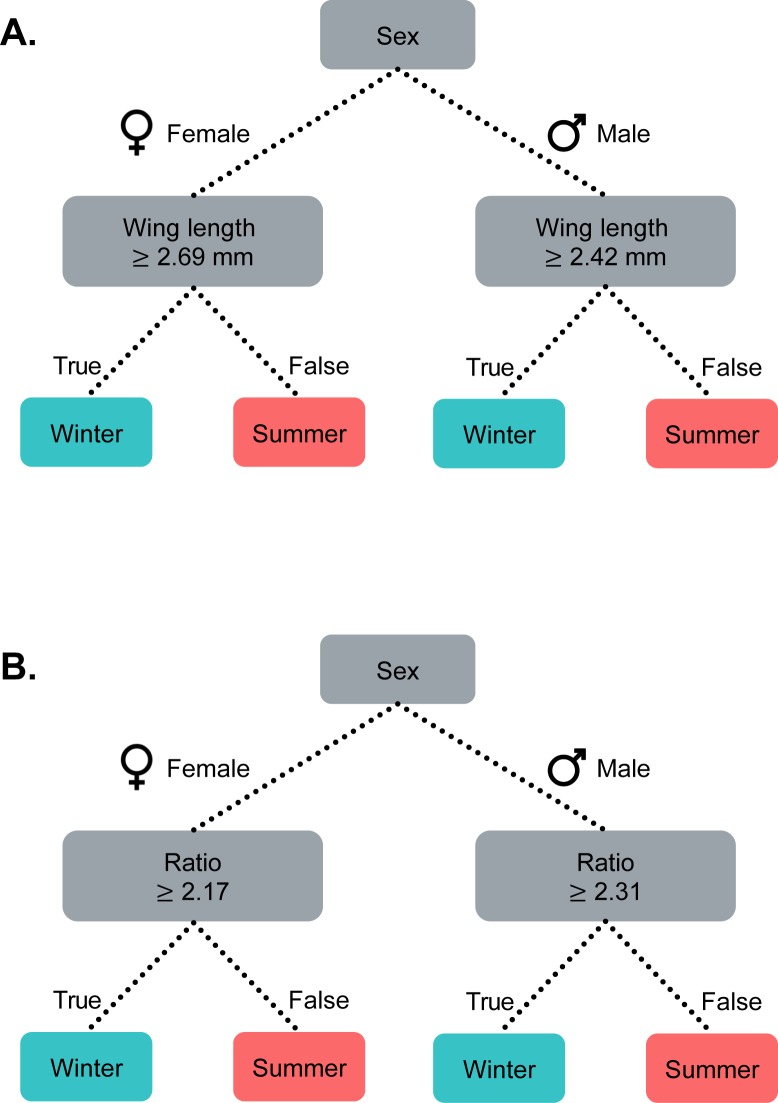
Classification tree model for differentiating winter and summer morphs *D*. *suzukii*. Classification criteria using wing length (A) and transformed ratio of wing length to hind length (B) for females and males.

**Fig 4:**
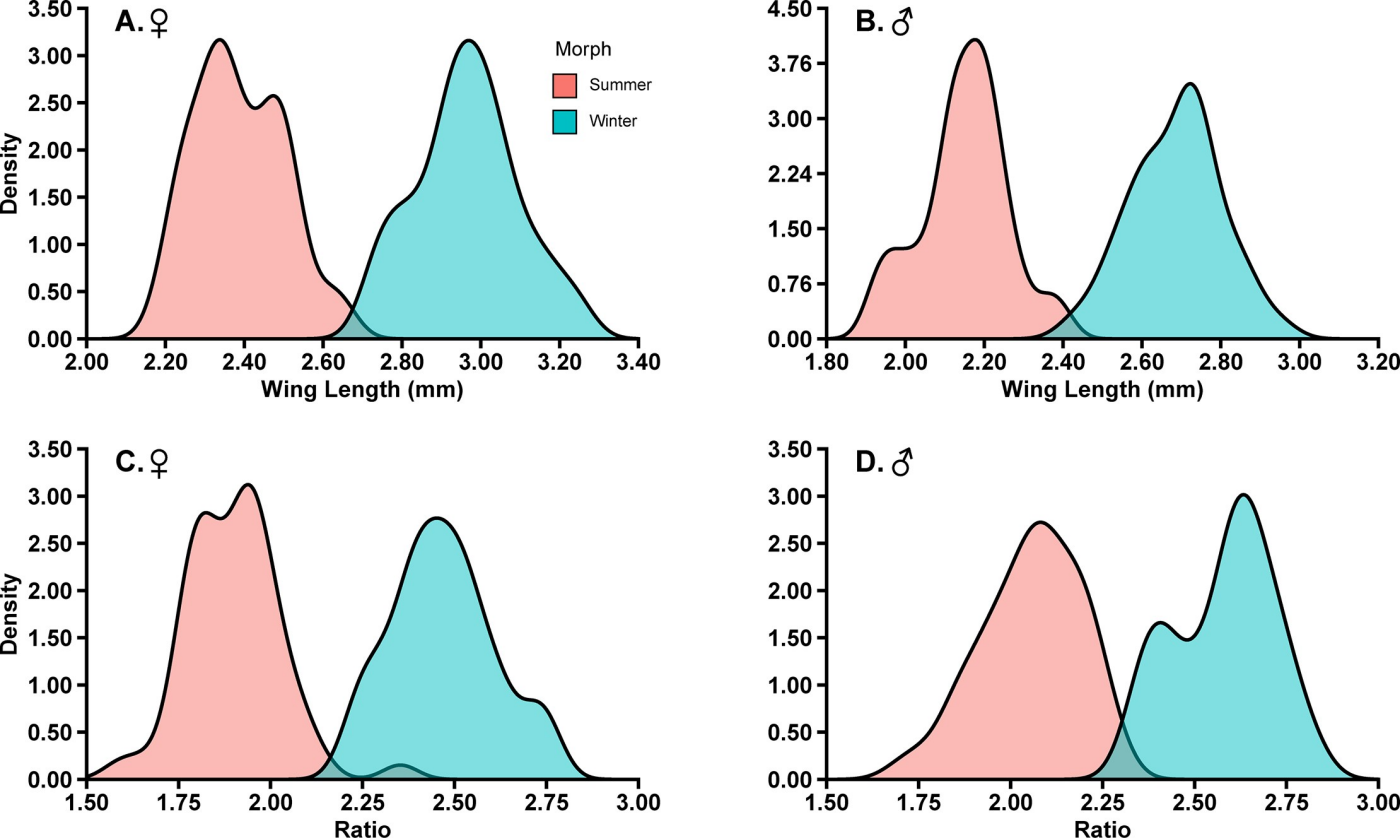
Density overlap for laboratory-reared *D*. *suzukii*. The percentage of density overlap based on wing length (A and B) and transformed ratio of wing length to hind tibia length (C and D) for female (A and C) and male (B and D).

Females and males with a wing length ≥ 2.69 mm and ≥ 2.42 mm, respectively, were classified as winter morphs (Table 2 and Fig 3A). From the cross-validation there was a total mean error rate of 0.10% and 1.82% for females and males, respectively (Table 2). There was a 2.00% density overlap in laboratory-reared *D*. *suzukii* for both female and male when using wing length alone as a morph predictor (Fig 4A and 4B).

For the transformed ratio of wing length to hind tibia length, females with a ratio ≥ 2.17, and males with a ratio ≥ 2.31, were classified as winter morphs (Table 2 and Fig 3B). From the cross-validation, there was a total mean error rate of 0.10% and 0.01% for females and males, respectively (Table 2). Using the transformed ratio as a morph predictor, the laboratory-reared *D*. *suzukii* had a density overlap of 2.00% and 4.00% for females and males, respectively (Fig 4C and 4D).

### Phenology of morph frequency in the field

**Fig 5:**
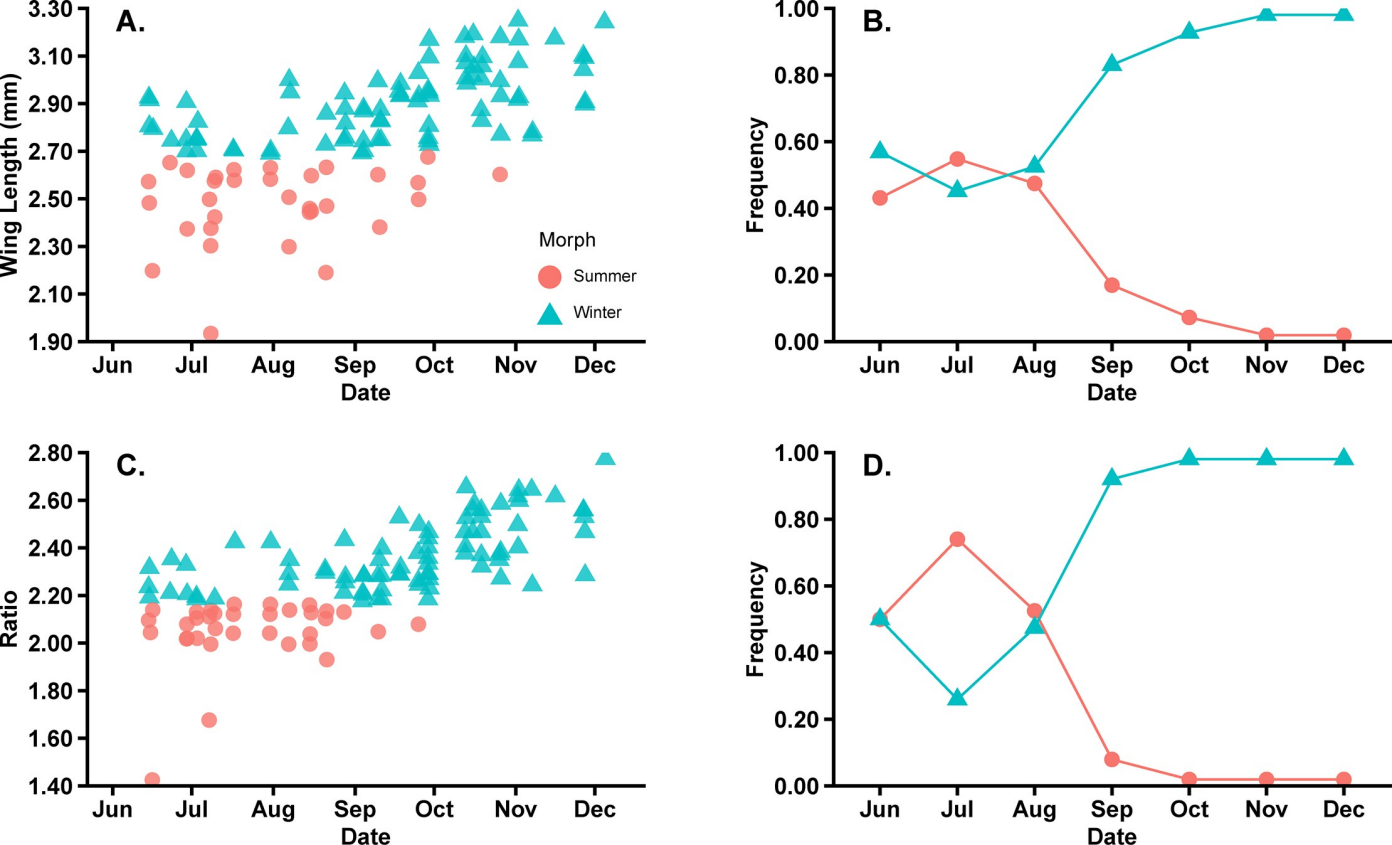
Predicted phenology of field caught female *D*. *suzukii*. Total of female *D*. *suzukii* morphs trapped from 2017–2018 (A and C) and the frequency (B and D) based on wing length (A and B) and transformed ratio of wing length to hind tibia length (C and D).

**Fig 6:**
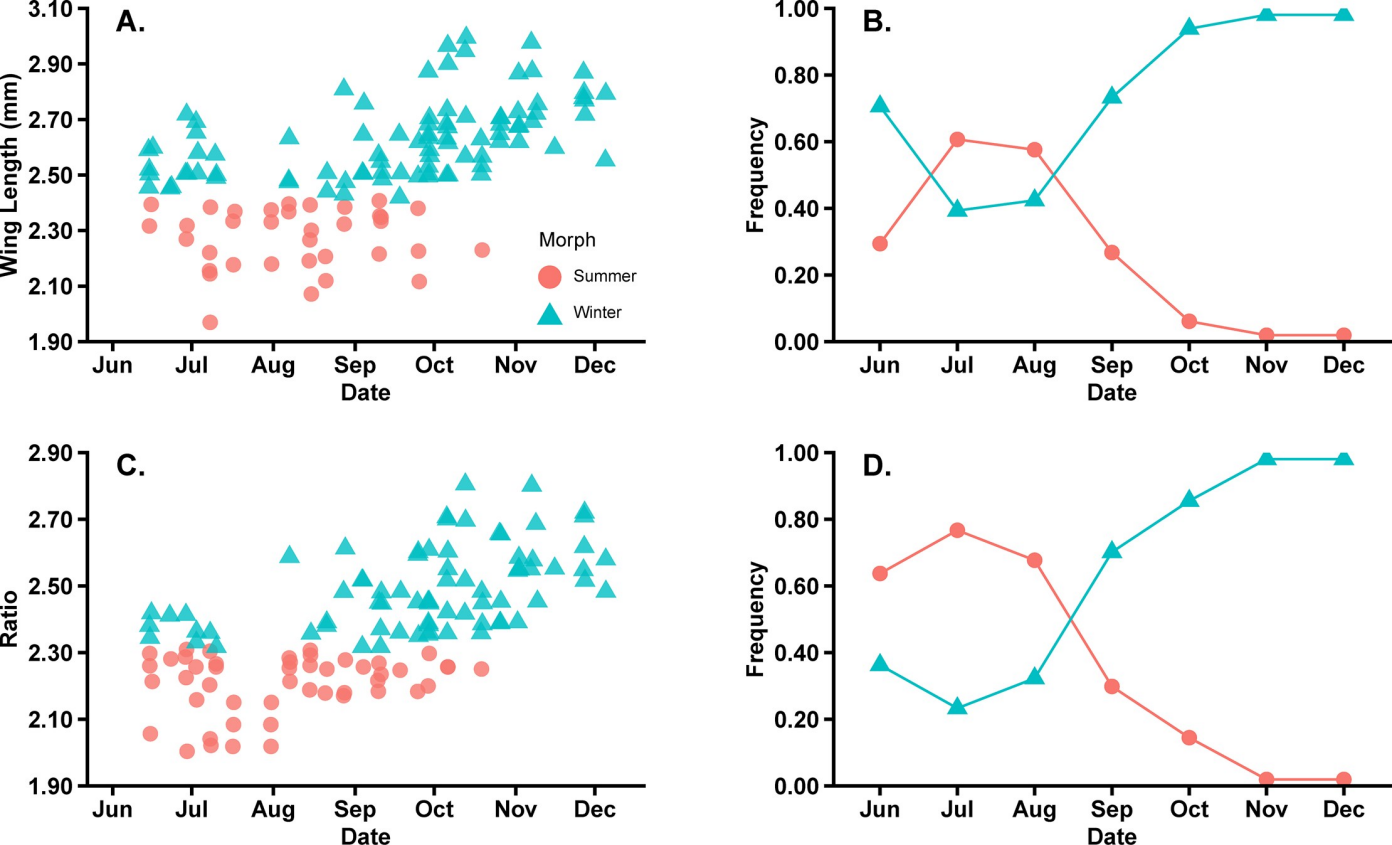
Predicted phenology of field caught male *D*. *suzukii*. Total of male *D*. *suzukii* morphs trapped from 2017–2018 (A and C) and the frequency (B and D) based on wing length (A and B) and transformed ratio of wing length to hind tibia length (C and D).

The results for each classification model (i.e., wing length and the transformed ratio of wing length to hind tibia length), were applied to field-caught *D*. *suzukii* to predict morph seasonal phenology (Figs 5 and 6). In Minnesota, *D*. *suzukii* were typically detected by mid-June (Figs 5 and 6). When using wing length to distinguish morphs, approximately 57.14% of females were identified as winter morphs in June (Fig 5B). In July, there is a decrease in identified winter morphs to 12.14%. From August to October, as the frequency of winter morph increases, approximately 94.44% of field-caught female *D*. *suzukii* were classified as winter morphs (Fig 5B).

When using the transformed ratio of wing length to hind tibia length to distinguish morphs, the pattern was similar to the wing length criteria, but at different frequencies. The transformed ratio criteria identified 50.00% of females as winter and summer morphs in June (Fig 5D). In July, the frequency of winter morphs identified decreased to 25.00%, and by October, 100% of female *D*. *suzukii* were classified as winter morphs.

The frequency of males caught in June that were identified as winter morphs using wing length was approximately 71.43% (Fig 6B). Similar to the female phenology results, there was a decline in the frequency in winter morphs during July and August, but the frequency increased to approximately 74.20–95.65% for September to October (Fig 6B). When using the transformed ratio to predict the morph seasonal phenology for males, the frequency of winter morphs identified in June was approximately 35.71% (Fig 6D). In July the frequency of winter morphs identified declined to approximately 13.50% before increasing throughout the remainder of the season (Fig 6D).

Of the two classification trees for females and males, a transformed ratio of wing length to hind tibia length may be the best for differentiating winter and summer morphs. Although the initial classification tree indicated that wing length was the best predictor for morph distinction, it is well known that diet can affect *D*. *suzukii* wing size [27]. We therefore elected to assess a ratio of wing length to hind tibia length as one approach to address the variability that can occur in field populations, but a transformation is required [58]. The initial results for the untransformed data are presented in S1 Table. The transformed ratio had similar results to wing length (Figs 5 and 6), but also had numerically higher levels of accuracy to determine male winter and summer morphs (Table 2). The level of overlap between known winter and summer laboratory-reared morphs was 2.00% for both females and males and the transformed ratio was higher for males of 4.00% (Fig 4). Regardless of having a higher density of overlap in males for a transformed ratio, the mean error for predicting winter and summer morphs was 0.01% compared to using the wing length where the mean error was 1.82% (Table 2).

## Discussion

A rigorous quantitative method for identifying *D*. *suzukii* morphs has not been developed and would be a valuable alternative tool to the identification protocol of winter and summer morphs. By comparing different morphometrics values we found that a transformed ratio of wing length to hind tibia length, for both females and males, was a reliable criterion for distinguishing winter and summer morphs for *D*. *suzukii* populations sampled in Minnesota. With this proposed criterion, *D*. *suzukii* morphs can potentially be determined using a standardized metric, but the method should be evaluated for other locations where *D*. *suzukii* is a concern. Our conclusions are based on the use of a classification tree to examine and detect patterns in the explanatory variables (i.e., wing length, wing width, and hind tibia length) that would best distinguish the morphs.

We used a classification tree model because the interpretation is straightforward relative to other analytical approaches [52,59]. Decision trees have been used previously in entomological research for determining predictor variables in both field and systematic studies [60–62]. For these reasons, a classification tree was appropriate for our research goal of developing a potential objective criterion that would differentiate *D*. *suzukii* morphs.

Thus far, an adult winter morph has not been knowingly caught in the early seasons in the North Central US, but this assessment was based on color as a distinguishing feature, which has important limitations. As mentioned previously, *D*. *suzukii* are typically trapped and contained in a liquid solution that could remain in the field for an extended period before the specimens are returned to the laboratory for examination. During this time, the specimen could be damaged making reliance on a color scale as an objective metric. Furthermore, a color scale can be subjective and arbitrary based on the observer [47,63]. Therefore, there was a need for developing an alternative technique for differentiating morphs. Using body measurements as a quantitative scale could be convenient as sclerotized body parts (i.e., wings and hind tibiae) are resistant to change in shape and size and can be stored for future research purposes.

We generated different classification methods that could be used to differentiate morphs, with each method changing our interpretations when applied to field populations. The classification analysis of laboratory specimens showed that wing length was the best predictor variable for differentiating winter and summer morphs, for both females and males. While wing length is a simple method because it only requires one body measurement, it may not be the most reliable due to a potential high variability of body size due to diet and/or temperature [27,64]. Therefore, a transformed ratio of wing length to hind tibia length was also examined as an alternative technique for determining morphs. Using the transformed ratio also dropped the mean error rate in males from 1.82% (wing length criteria) to 0.01% (Table 2). The further reduction in error rate would be useful for those identifying *D*. *suzukii* that are present in the overlapping areas where differentiating morphs may be most difficult to distinguish (Fig 4D).

When predicting the phenology of *D*. *suzukii* morphs using both wing length and transformed ratio of wing length to hind tibia length, our results showed that female (Fig 5) and male (Fig 6) winter morphs are present in June. While this result has not been the case for other North American surveys, trapping data over time from other regions suggest that winter morphs are present early in the calendar year. For example, Panel et al. [33] documented the phenology of winter and summer morphs in the Netherlands, noting that winter morphs were detected year-round with a rapid decline in June when that would be assumed the transition period to summer morphs. Additionally, Thistlewood et al. [31] trapped *D*. *suzukii* from January to April in the Pacific Northwest, USA, suggesting some of the trapped flies may be winter morphs. Our data may provide evidence for the earliest detection of a *D*. *suzukii* winter morph in Minnesota and the upper Midwest region, based on the use of wing length or the transformed ratio of wing length to hind tibia length as morph indicators. This is a unique finding because in Wisconsin, an adjacent Midwest state, the first winter morph was detected in August based on body pigmentation [44]. As Wisconsin and Minnesota can have similar temperature profiles, these contrasting results further demonstrate the need for creating a definitive strategy for differentiating winter and summer morphs to understand *D*. *suzukii* seasonal phenology.

*Drosophila suzukii* body size can vary due to diet. Additional research recording the effects of different host plants on the body and wing sizes of *D*. *suzukii* morphs, for multiple populations, might improve the classification tree model to better represent flies in the field, especially if information on differential host availability and use are known for different locations. Information about different host plants may be particularly important because Jaramillo et al. [64] did not find differences between artificial diet and blueberry affecting wing length for *D*. *suzukii* females and males, while Wallingford and Loeb [27] found differences between artificial diet and raspberries. This further signifies the phenotypic plasticity of this species.

Our classification model demonstrates an alternative method for differentiating winter and summer morph *D*. *suzukii*. Using this quantitative scale for Minnesota indicated that winter morphs can occur at a high frequency early in the season, which has not been found in other studies within the northern Midwest states. Future studies should compare the color-based system and the quantitative scale on field-caught *D*. *suzukii* and to analyze the phenology of winter and summer morphs based on the two different identification techniques.

Of the two different classification models, we suggest that the transformed ratio of wing length to hind tibia length may be the most useful, robust morphometric for differentiating winter and summer morphs. While wing length was selected as the best morphological characteristic from the classification tree analysis, there is a chance this would underestimate winter morph’s wing length due to not knowing the effects different crop hosts have on *D*. *suzukii* wing size in the field. It is often unknown what host plants are being utilized by *D*. *suzukii* field populations, thus using wing length alone, though a simple measurement, would not fully address size variability due to host availability.

The wing length to hind tibia length ratio would potentially correct for the size variation of field-caught and laboratory-reared *D*. *suzukii*. However, a transformation is required, as simple ratios rarely control for size variation ([58], S1 Table). The transformed ratio of wing length to hind tibia length had a reduced error rate (Table 2) and produced similar results as the wing length criteria in terms of predicted phenology (Figs 5 and 6). Given this outcome, to identify field-caught *D*. *suzukii* morphs, the ratio of wing length to hind tibia length should be transformed by subtracting 1.008 (females) or 0.762 (males) from the wing length and dividing the following value by the hind tibia length. Female and male *D*. *suzukii* with a transformed ratio of ≥ 2.17 and ≥ 2.31, respectively, would then be identified as winter morphs (Fig 3B).

There are several other avenues of research that follow from this study. For instance, previously stored *D*. *suzukii* samples from multiple locations could be examined to understand the phenology of winter versus summer morphs at different latitudes, and thus further our understanding of winter survival strategies of *D*. *suzukii*. Early-caught *D*. *suzukii* morph identification could change if the flies were identified based on a quantitative scale instead of color schemes alone. While local overwintering is certainly a valid hypothesis for winter morph production in temperate latitudes, there are other alternative explanations that have not been adequately investigated. For example, while it has generally been assumed that the darker coloration and larger body size in *D*. *suzukii* is to aid in overwintering, many insect species are known to produce variants that have larger wings for migratory purposes [65–67]. The testing of alternative hypotheses for winter morph production in *D*. *suzukii*, which may not necessarily be mutually exclusive, is vital to gaining a complete understanding of the population dynamics of this devastating fruit pest.

## Conclusion

*Drosophila suzukii* is highly phenotypically plastic, and their color and size variation can change depending on environmental cues. However, until recently, research has been limited with regard to understanding the adaptive benefits of winter versus summer morphs, and how to distinguish them in a systematic fashion. Reliance on color for differentiating *D*. *suzukii* has been suggested as an unreliable indicator for morphs [47,63], and further supports the need of a quantitative metric scale. In this paper, we test a specific morphometric approach to determine winter and summer morphs focused primarily on wing length and the transformed ratio of wing length to hind tibia length. This method was developed using a predictive statistical analysis and eliminates the bias that may be apparent when using a color scale. Research continues to expand on understanding *D*. *suzukii* biology and phenology to improve management practices. However, scientists struggle with finding a definitive method for identifying winter and summer morphs. We conclude that our study provides a potential practical technique for differentiating morphs using body and wing measurements and have identified future research directions for its field implementation.

## Supporting information

S1 Table Summary results of the classification tree using the ratio of wing length to hind tibia to differentiate winter morphs from summer morphs. (DOCX)

S1 Fig Laboratory-reared mean wing and hind tibia lengths (mm) for known female (A) and male (B) winter and summer morphs of *D*. *suzukii*, compared with field-caught females and males.
(TIF)

S2 Fig Residual plot of laboratory-reared known summer morphs for females (A) and males (B).
(TIF)
