# Supplementary material for: Morphometric criteria to differentiate Drosophila suzukii (Diptera: Drosophilidae) seasonal morphs
Source: PLoS One. 2020 Feb 6;15(2):e0228780. doi: 10.1371/journal.pone.0228780 (PMC7004555; doi:10.1371/journal.pone.0228780)
Supplement: S1 Table — (DOCX) [file pone.0228780.s001.docx]

**S1 Table:** Summary results of the classification tree using the ratio of wing length to hind tibia to differentiate winter morphs from summer morphs.

| **Sex** | **Average statistics for 500 classification simulations** | | | | | | |
| --- | --- | --- | --- | --- | --- | --- | --- |
|  | **Ratio cutoff value** | | |  | **Error (%)** | | |
|  | **Mean** | **Min** | **Max** |  | **Mean** | **Min** | **Max** |
| Female | 3.48 | 3.40 | 3.55 |  | 7.24 | 0.00 | 24.14 |
| Male | 3.36 | 3.31 | 3.49 |  | 8.24 | 0.00 | 25.00 |

The classification was built using measurements from laboratory-reared known morphs of *Drosophila suzukii*, where 70% of data were used for training and 30% of the data were used for validation. This process was bootstrapped 500 times and the results were averaged.
