# Supplementary figures and images for: Morphometric criteria to differentiate Drosophila suzukii (Diptera: Drosophilidae) seasonal morphs
Source: PLoS One. 2020 Feb 6;15(2):e0228780. doi: 10.1371/journal.pone.0228780 (PMC7004555; doi:10.1371/journal.pone.0228780)

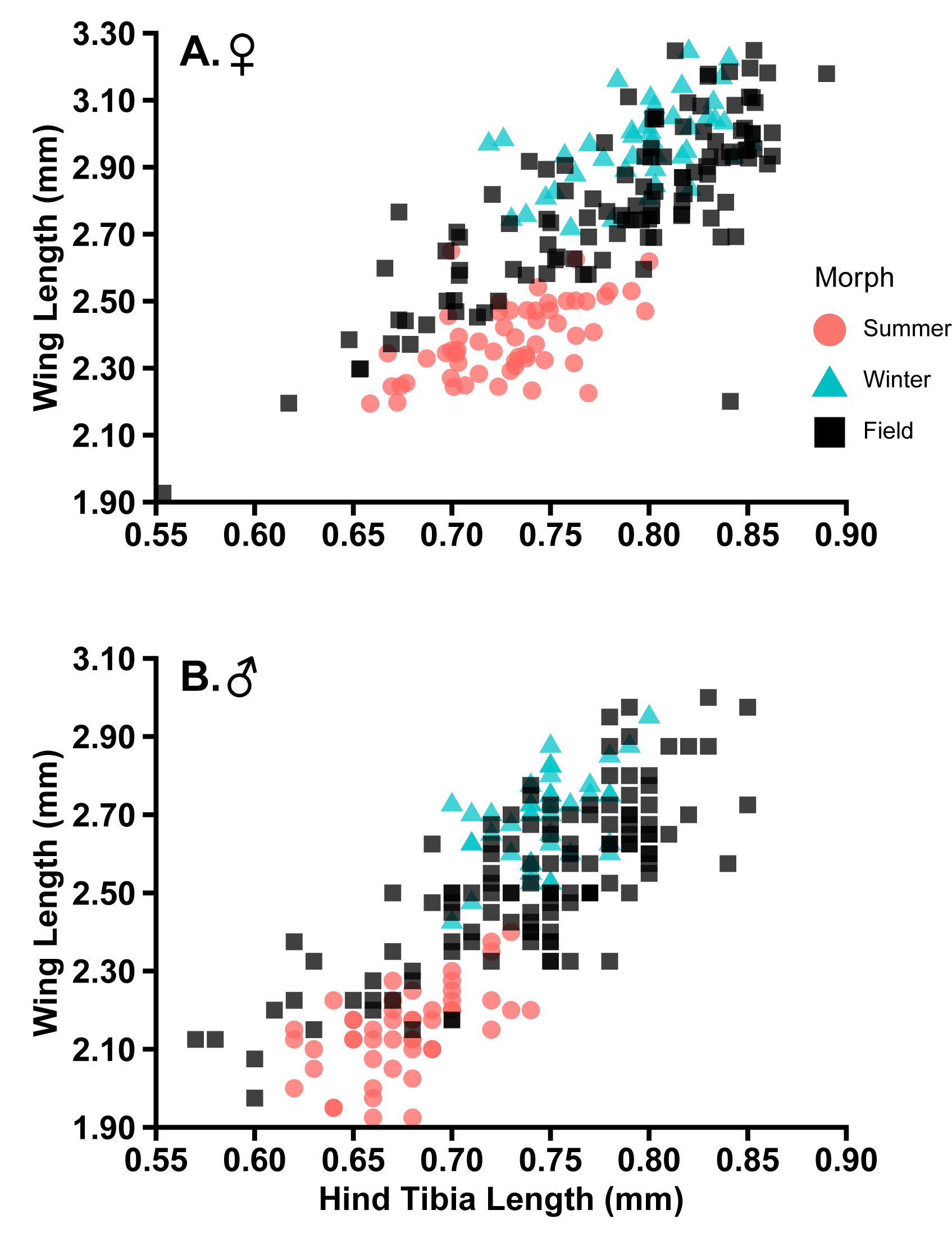

Supplement: S1 Fig — Laboratory-reared mean wing and hind tibia lengths (mm) for known female (A) and male (B) winter and summer morphs of D. suzukii, compared with field-caught females and males. (TIF) [file pone.0228780.s002.tif]

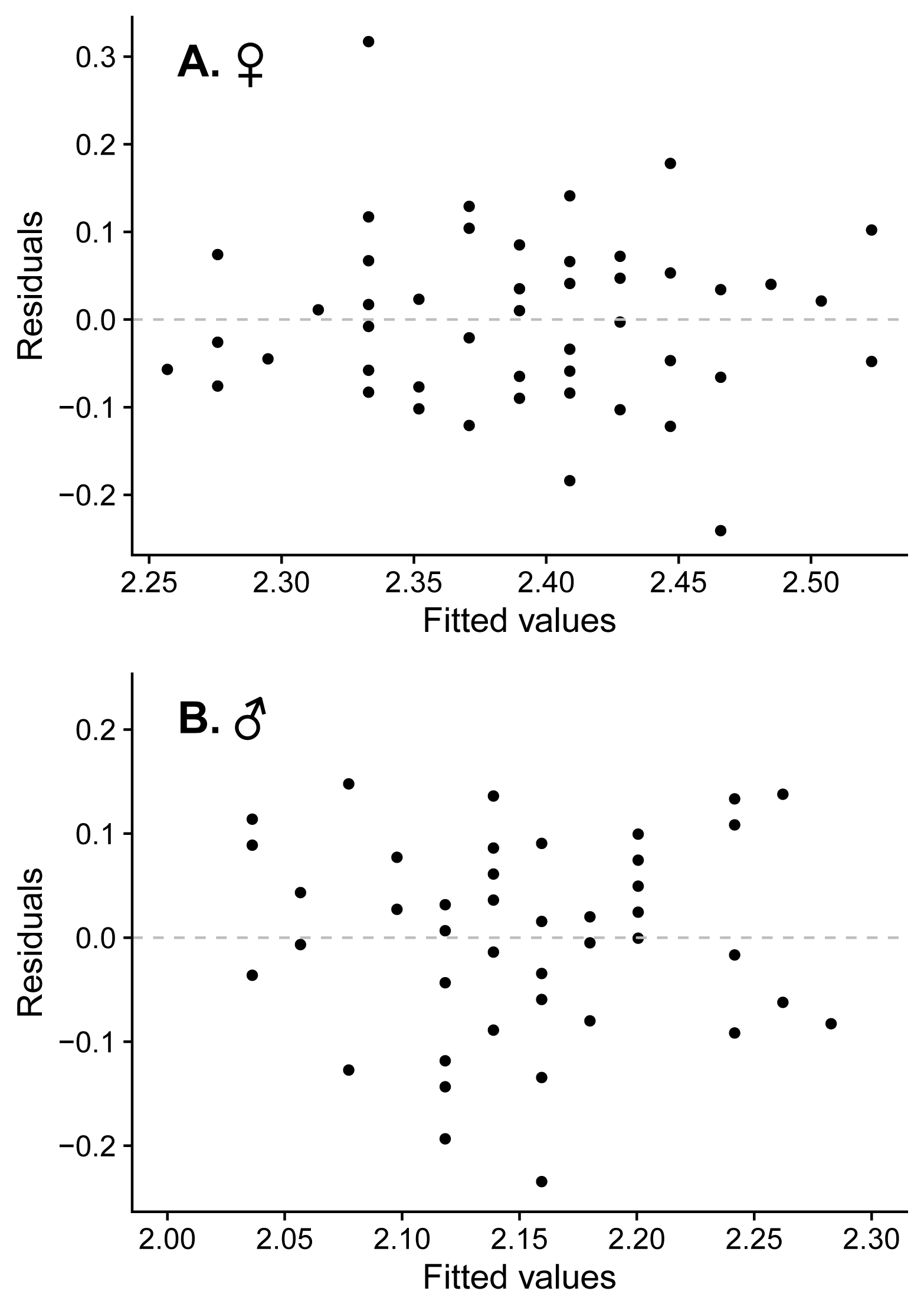

Supplement: S2 Fig — Residual plot of laboratory-reared known summer morphs for females (A) and males (B). (TIF) [file pone.0228780.s003.tif]
